# EEG-FCV: An EEG-Based Functional Connectivity Visualization Framework for Cognitive State Evaluation

**DOI:** 10.3389/fpsyt.2022.928781

**Published:** 2022-07-11

**Authors:** Hong Zeng, Yanping Jin, Qi Wu, Deng Pan, Feifan Xu, Yue Zhao, Hua Hu, Wanzeng Kong

**Affiliations:** ^1^College of Computer and Technology, Hangzhou Dianzi University, Hangzhou, China; ^2^Key Laboratory of Brain Machine Collaborative Intelligence of Zhejiang Province, Hangzhou Dianzi University, Hangzhou, China; ^3^School of Information Science and Technology, Hangzhou Normal University, Hangzhou, China

**Keywords:** EEG, functional connectivity, Comprehensive, brain cognitive function, visualization

## Abstract

Electroencephalogram (EEG)-based tools for brain functional connectivity (FC) analysis and visualization play an important role in evaluating brain cognitive function. However, existing similar FC analysis tools are not only visualized in 2 dimensions (2D) but also are highly prone to cause visual clutter and unable to dynamically reflect brain connectivity changes over time. Therefore, we design and implement an EEG-based FC visualization framework in this study, named EEG-FCV, for brain cognitive state evaluation. EEG-FCV is composed of three parts: the Data Processing module, Connectivity Analysis module, and Visualization module. Specially, FC is visualized in 3 dimensions (3D) by introducing three existing metrics: Pearson Correlation Coefficient (PCC), Coherence, and PLV. Furthermore, a novel metric named Comprehensive is proposed to solve the problem of visual clutter. EEG-FCV can also visualize dynamically brain FC changes over time. Experimental results on two available datasets show that EEG-FCV has not only results consistent with existing related studies on brain FC but also can reflect dynamically brain FC changes over time. We believe EEG-FCV could prompt further progress in brain cognitive function evaluation.

## 1. Introduction

More and more studies prove a strong association between brain cognitive function and neural connectivity in different brain regions (1–3). An EEG-based brain connectivity analysis provides much useful and meaningful information to reflect the dynamic changes in brain neural activities, and an interpretation of the changes in cognitive functions as well (4–7). Therefore, it is of vital importance to design a friendly human-computer interaction visualization framework to better reflect the connectivity and its dynamical changes in the brain for evaluation of cognitive states, which can not only promote the understanding of EEG signals but also assist better performing the relevant analysis of brain cognitive function. In general, there are three kinds of connectivity: anatomical, effective, and functional (8). Since the definition of anatomical connectivity is not yet clear, and some fake connections may be involved in the effective connection, we mainly focus on functional connectivity (FC) in our designed framework.

Functional connectivity is defined as the temporal correlation between spatial remote neurophysiological events (9) and is essentially employed to measure the degree of dependence or correlation between signals collected by relevant techniques, such as functional magnetic resonance imaging (fMRI) (10–12), diffusion tensor imaging (DTI) (13) and EEG (14, 15), etc. Compared with fMRI and DTI, EEG has millisecond-level time resolution, which makes it more suitable for visualizing brain neural activities, and easier to reflect changes in brain activity in the temporal dimension (16) as well.

In the past decades, although many teams (17–21) have devoted themselves to the development of similar toolkits for analysis and visualization of brain FC, there are still some issues to be addressed. First, existing related toolkits are not platform-independent, most of which such as ELAN (21), and EEGNET (19), are embedded in some platform software like MATLAB (Math works, Inc.), making them incapable of running independently. Second, those toolkits lack effective algorithms to eliminate the problem of visual clutter. Finally, most of them can only perform a kind of static FC analysis, so they cannot reflect the dynamic changes of EEG cognitive state.

Therefore, in this article, we design an EEG-based FC visualization framework (EEG-FCV) to visualize brain connectivity between different channels, different brain regions, as well as left and right hemispheres, respectively, so as to better estimate the brain's cognitive functions and their dynamic changes. Besides, we propose a novel brain connectivity metric, named Comprehensive, to eliminate visual clutter.

The main contributions in this study are involved in the following three aspects:

- We design and implement a 3D software framework known as EEG-FCV which is completely independent of platforms, for the visualization of brain FC and evaluation of cognitive states.
- We propose Comprehensive as a novel metric of brain FC to efficiently solve the problem of visual clutter.
- We take advantage of the high time resolution of EEG to realize FC dynamic visualization, so as to provide some assistance with an analysis of the evolution of brain cognitive activities.

## 2. Related Study

### 2.1. EEG-Based FC Evaluation Methods

Among those methods for EEG-based evaluation of brain FC, the most representatives usually include Correlation, Coherence, and Phase Locking Value (PLV). For example, Risqiwati et al. (22) performed EEG-based FC analysis with Pearson Correlation metric in different mental states and found obvious FC effect in Beta (13–30 Hz) and Theta (4–7 Hz) frequency bands. In Duffy and Als (23). adopted Spectral Coherence to analyze brain FC of children with autism and confirmed the changes in connectivity with the coherence metric. La Rocca et al. (24) further fused spectral coherence-based connectivity between different brain regions as a possibly viable biometric feature, concluding that Coherence is a useful metric in the evaluation of brain FC. In addition, PLV-based metric is also used to perform FC analysis to study the difference between the Supplementary Motor Area (SMA) and Primary Motor Area (M1) when performing motor imagery tasks of left/right hand movement (25). The experimental results showed that PLV is one of the robust features to distinguish different motor imagery states. Sadaghiani et al. (26) also concluded that alpha band (8–13 Hz) phase synchrony was linked to neural structures underpinning phasic control of alertness and task requirements.

### 2.2. EEG-Based FC Visualization Applications

Nowadays, many research teams have developed various EEG FC visualization tools. Hassan et al. (19) developed an EEGNET plug-in application embedded in MATLAB for the analysis of functional brain networks. EEGNET could perform EEG preprocessing and carry out EEG-based analysis of brain FC among different surface electrodes. To study the synchronization patterns of different EEG acquisition channels, Alba et al. (17) designed a time-frequency-topography visualization system based on various techniques, such as time-frequency decomposition and Bayesian approach, to perform various synchrony measurements. In order to reduce the visual clutter of FC visualization produced by coherence measures from multichannel EEG, Ten Caat et al. (18) optimized the way of graphical layout through a maximal clique-based (MCB) method and a watershed-based (WB) method with a better visualization effect. ELAN is a freely available software package, developed by the Brain Dynamics and Cognition Laboratory in Lyon (21), for the analysis of scalp and intracranial EEG. ELAN could provide phase synchronization analysis of EEG and visualization of topographic mapping. Peyk et al. (20) provided a MATLAB-based EMEGS toolkit that can visualize data as 3D projections of multiple models, including simple sphere models, realistic head shapes, and realistic brain shapes.

Although many EEG-based FC analysis and visualization tools have been developed, most of them have to be embedded in some platform software such as MATLAB as the plug-in toolboxes, so they are difficult to avoid visual clutter and cannot reflect the dynamic changes in brain connectivity. All these factors have hindered the further promotion of these existing tools.

## 3. Framework of EEG-FCV

**Figure 1:**
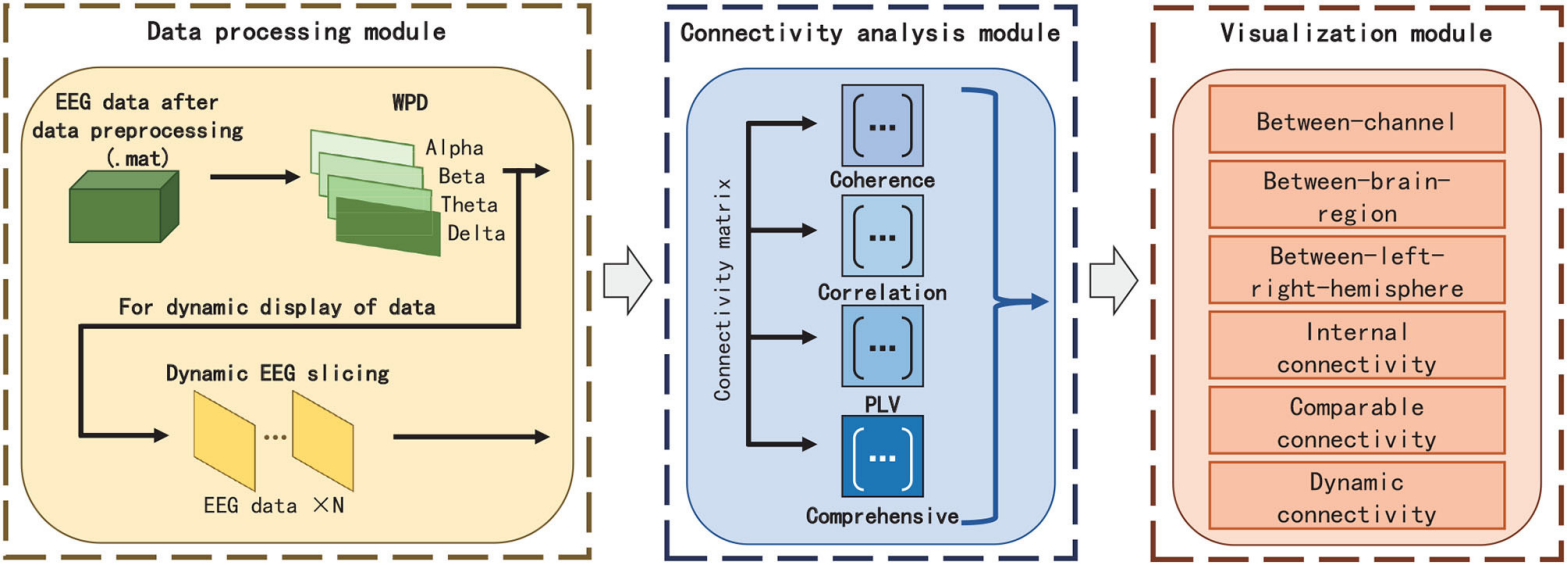
Overall framework of EEG-FCV.

**Figure 2:**
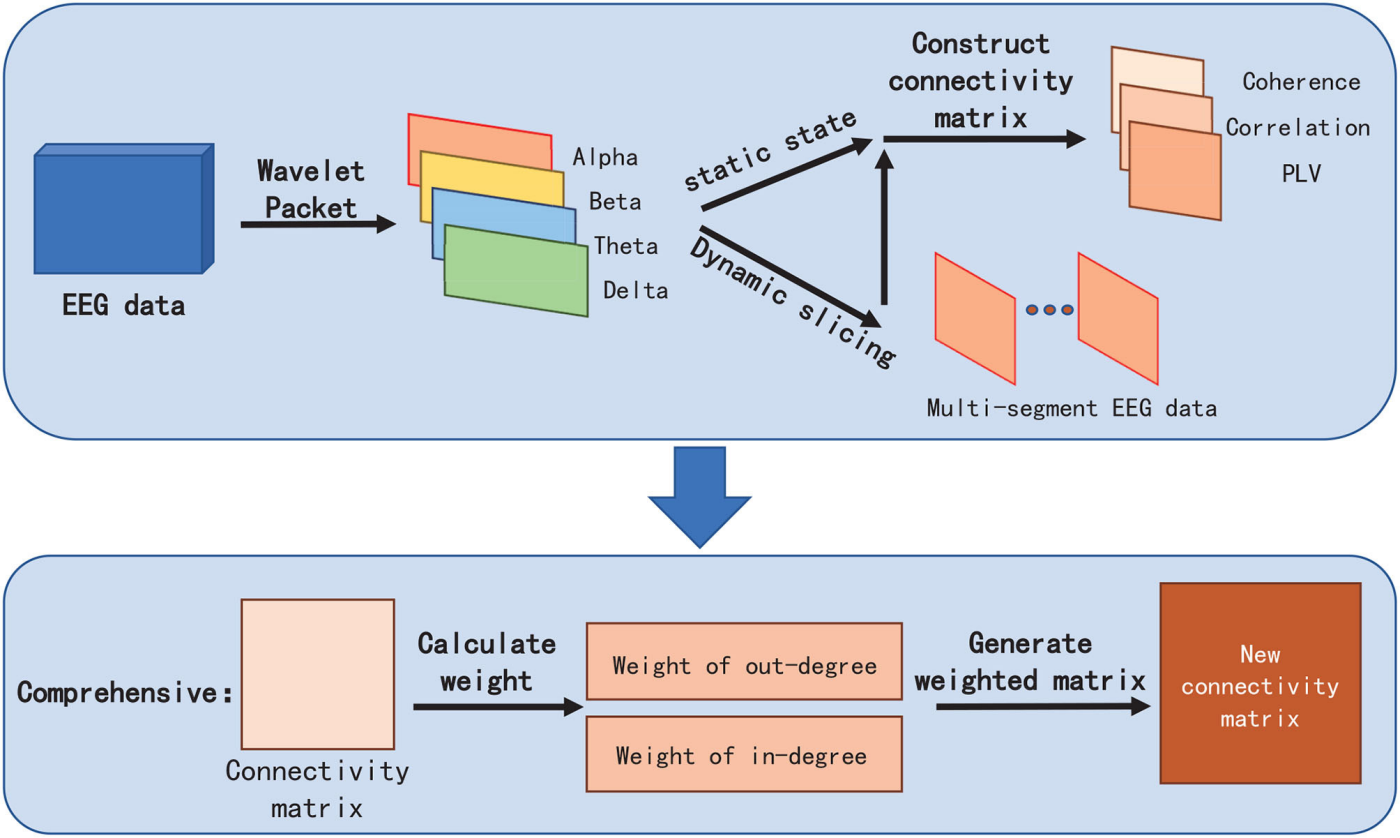
Work flow of EEG-FCV.

The framework and work flow of EEG-FCV are shown in Figures 1, 2, respectively. EEG-FCV is composed of three modules: Data Processing Module, Connectivity Analysis module, and Visualization module. The raw EEG data first undergoes preliminary preprocessing, including artifact removal and down-sampling. Then the new-generated data file in .mat format is sent into the Data Processing Module.

### 3.1. Data Processing Module

Usually, EEG signals of the Alpha frequency band (8–13 Hz) in the occipital lobe and parietal lobe appear and play a major role when someone is awake, quiet, and eye-closed. When a person is in a state of concentration, thinking, alertness or anxiety, EEG signals of the Beta frequency band in the frontal lobe are strongly correlated with brain cognitive activities. EEG signals in the Theta frequency band are more active near the Fz lobe when someone is in meditation, drowsiness, hypnosis, or sleep state. EEG signals in the Delta frequency band (1–4 Hz) are dominated during sleeping (27–29). Therefore, we can infer the cognitive function by observing the connectivity and its changes in different frequency bands.

Therefore, in Data Processing Module, we first extract EEG features by adopting Wavelet Packet Decomposition (WPD) in frequency bands of Alpha, Beta, Theta, and Delta, respectively. EEG feature vectors in these four frequency bands can be directly used as the input of the Connectivity Analysis module for static visualization. Regarding the dynamic display of FC visualization, we perform slicing with a fixed time interval according to different feature labels in EEG to obtain multiple EEG feature vectors with equal length and then send those features to the Connectivity Analysis module to calculate FC metrics for visualization.

### 3.2. Connectivity Analysis Module

In the Connectivity Analysis module, we not only embed three commonly used FC metrics(PCC, Coherence, and PLV) but also propose a novel FC metric named Comprehensive to extract the connection weight and highlight the strength of the connectivity between EEG channels, so as to facilitate the elimination of vision clutter brought by commonly used FC metrics algorithms.

#### 3.2.1. Pearson Correlation Coefficient

Given two time series of signals *x* = {*x*_1_, *x*_2_, ⋯ , *x*_*i*_, ⋯ , *x*_*n*_} and *y* = {*y*_1_, *y*_2_, ⋯ , *y*_*i*_, ⋯ , *y*_*n*_} (*x*_*i*_ and *y*_*i*_ are the sample points with an index of *i*), *PCC* *r* is in statistics the measure of linear correlation between *x* and *y*. It is the ratio between the covariance of the two signals and the product of their SDs. Thus, *PCC* is essentially a normalized measurement of the covariance, and the result has a value between −1 and 1. Thus, for *n* paired data {*x*_*i*_, *y*_*i*_} in *x* and *y*, *PCCr* is defined as:


(1) $$\begin{array}{r} r=\frac{\sum_{i=1}^{n}\left(x_{i}-\bar{x}\right)\left(y_{i}-ȳ\right)}{\sqrt{\sum_{i=1}^{n}{\left(x_{i}-\bar{x}\right)}^{2}}\sqrt{\sum_{i=1}^{n}{\left(y_{i}-ȳ\right)}^{2}}} \end{array}$$

where $\bar{x}$ and ȳ are the sample means which equals to $\frac{1}{n}\overset{n}{\underset{i=1}{\sum }}x_{i}$ and $\frac{1}{n}\overset{n}{\underset{i=1}{\sum }}y_{i}$, respectively. The greater the absolute of *r*, the stronger the correlation.

#### 3.2.2. Coherence

Coherence, also called spectral coherence in signal processing, is commonly used to estimate the power between input and output of a linear system. The coherence *Coh*_*xy*_ between *x* and *y* can be calculated as Equation 2:


(2) $$\begin{array}{r} Coh_{xy}=\frac{|p_{xy}\left(f\right){|}^{2}}{p_{xx}\left(f\right)\cdot p_{yy}\left(f\right)} \end{array}$$

where *p*_*xy*_(*f*) is the cross-spectral density at a frequent band *f* between *x* and *y*, and *p*_*xx*_(*f*) and *p*_*yy*_(*f*) are the auto-spectral density of *x* and *y*, respectively. The magnitude of the spectral density is denoted as |*p*|.

*Coh*_*xy*_ always satisfies 0 ≤ *Coh*_*xy*_ ≤ 1. Similar to *PCC* *r*, if *Coh*_*xy*_ is closer to 1 at frequent band *f*, there exists a stronger relationship between *x* and *y*.

#### 3.2.3. Phase Locking Value

Phase Locking Value, also known as phase synchronization index, is a phase-based FC method, which actually measures the phase difference between two channels. *PLV* between *x* and *y* can be written as:


(3) $$\begin{array}{r} PLV=|n^{-1}\overset{n}{\underset{t=1}{\sum }}e^{i\left(\phi_{xt}-\phi_{yt}\right)}| \end{array}$$

where ϕ_*xt*_ and ϕ_*yt*_ represent the phase angles of *x* and *y*, respectively at time *t*. The range of *PLV* is [0,1]. The larger the value *PLV*, the stronger the phase synchronization between *x* and *y*.

Phase Locking Value can be used to investigate task-induced changes in long range synchronization of neural activity from EEG data, and statistics can be argued to be a proxy for connectivity. Intuitively, if the EEG signal in two channels (electrodes) during an experimental condition rises and falls together more than a baseline value, then there is more synchronization, or loosely speaking, enhanced connectivity between these two electrodes. If it is less than the baseline value, there is de-synchronization, or loosely speaking, decreased connectivity between the two electrodes.

Although the above three metrics are commonly used for FC analysis, they do not consider the co-variation in the power of the EEG signal between two electrodes and are more sensitive to the volume conduction effect. However, it might not only lead to visual clutter due to the smaller difference between the three metrics in connectivity strength but also be difficult to highlight the connectivity to find an optimal connection between channels in the brain network.

#### 3.2.4. Our Proposed Metric of Comprehensive

**Figure 3:**
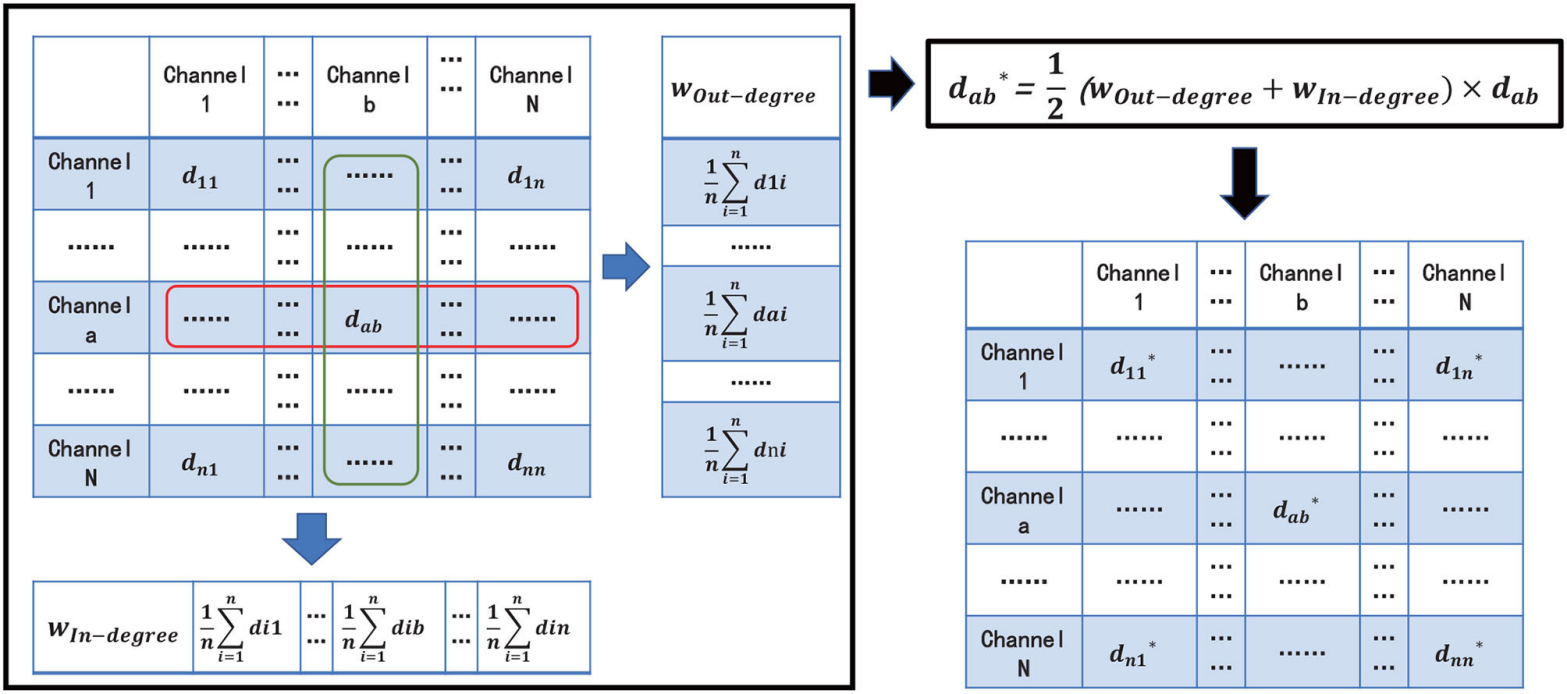
Comprehensive EEG-FCV.

To better reflect the connectivity difference between channels, and eliminate the vision clutter, we propose a novel measure metric, named *Comprehensive*. The core idea of *Comprehensive* is to assign a weight for FC visualization to the edge between two channels (also termed as nodes in the brain network) in the brain network by respectively calculating the degrees of the two channels (Figure 3). *Comprehensive* can not only show the difference in the connectivity strength between channels and, thus, find the key connectivity among them but also can efficiently eliminate the visual clutter. In detail,

First, for the *a*^*th*^, *b*^*th*^ channels *C*_*a*_ and *C*_*b*_ in channel set {*C*_1_, *C*_2_, ⋯ , *C*_*n*_}, *a, b*∈{1, 2, ⋯ , *n*}, we define, respectively the in-degree weight *w*_*In*−*degree*_ and the out-degree weight *w*_*Out*−*degree*_ of channel *C*_*a*_ in Equations 4 and 5 as the average connectivity of the number of head adjacent channel *C*_*i*_, and tail end adjacent channel *C*_*j*_ to channel *C*_*a*_, respectively :


(4) $$\begin{array}{r} w_{In-degree}=\frac{1}{n}\overset{n}{\underset{i=1}{\sum }}d_{ia} \end{array}$$

(5) $$\begin{array}{r} w_{Out-degree}=\frac{1}{n}\overset{n}{\underset{j=1}{\sum }}d_{aj} \end{array}$$

where *i, j* ∈{1, 2, ⋯ , *n*} are the subscripts of *i*^*th*^ channel *C*_*i*_ and *j*^*th*^ channel *C*_*j*_. *d*_*ia*_ represents one metric of *PCC* *r*, *Coh*_*ia*_, and *PLV* from channel *C*_*i*_ to *C*_*a*_. *d*_*aj*_ is similar to *d*_*ia*_.

Then, *Comprehensive* $d_{ab}{}^{*}$ can be denoted as:


(6) $$\begin{array}{r} d_{ab}^{*}=\frac{1}{2}\left(w_{Out-degree}+w_{In-degree}\right)\times d_{ab} \end{array}$$

Similar to *PCC, Coherence*, and *PLV*, we need to normalize $d_{ab}^{*}$ in line with different metrics chosen by *d*_*ab*_. For example, if we choose *PCC* or *PLV* as *d*_*ab*_, $d_{ab}^{*}$ will be between 0 and 1, while *Coherence* as *d*_*ab*_, $d_{ab}^{*}$ will lie between −1 and 1.

### 3.3. Visualization Module

#### 3.3.1. Basic Concepts

The static/dynamic visualization is realized in the Visualization module. In this module, six FC visualizations, including between-channel, between-brain-region(between-BR), between-left-right-hemisphere(between-L/RH), internal-connectivity, comparable connectivity, and dynamic connectivity are designed to show FC effects in different situations.

Herein, between-channel FC visualization means to visualize the connectivity between two different channels. Between-BR visualization is to show the connectivity between two different BRs distributed in line with the international 10–20 system, which are frontal lobe, parietal lobe, occipital lobe, temporal lobe, and central zone, respectively. Correspondingly, the connectivity between two different hemispheres is shown in between-L/RH visualization. Internal connectivity visualization is to reveal the connectivity results between channels either in the same BR or in the same L/RH. In addition, EEG-FCV also provides comparable FC visualization of different cognitive states, so as to facilitate analyzing FC differences for various psychological activities. Finally, as a special function of EEG-FCV, the FC changes of EEG over time are realized in Dynamic Visualization, which helps researchers to better observe the connectivity changes, thereby assisting in the study of relevant potential neuropsychological activities.

#### 3.3.2. 3D Display in Different Visualization Levels

**Figure 4:**
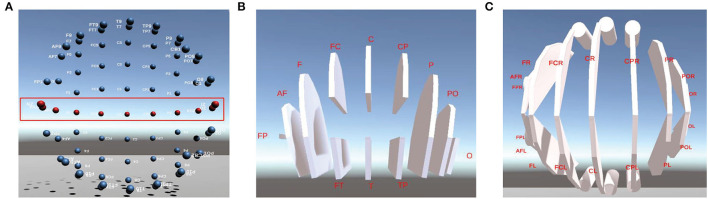
Three ways of Visualization. **(A)** is the model of between-channel, **(B)** is the model of between-BR, and **(C)** is the model of between-L/RH.

3ds Max software (Autodesk Company, USA) is employed as the development tool to construct our 3D visualization module. There are three different levels of visualization, which are between-channel (Figure 4A), between-BR (Figure 4B), and between-L/RH (Figure 4C).

Between-channel level of 3D visualization is designed and implemented in terms of the 81-channel's 3D coordinates of the standard 10–20 lead provided by EEGLab. For better visualization, we mark those channels in the central zone of the cerebral cortex (such as Nz-Iz) in red and those in other areas in blue, respectively, as shown in Figure 4A. In order to show 3D visualization of the between-BR level, we first connect the 3D coordinates of all the channels in the same BR to generate a polyhedron. According to this method, a total of *12* visualization levels of different BRs marked in white is generated. Similarly, the 3D visualization of between-L/RH levels can also be achieved, as shown in Figures 4B,C, respectively.

### 3.4. Connectivity Visualization

In EEG-FCV, the connectivity strength is represented by different colors, transparency, and thickness of connection lines. The golden-and-red lines indicate the connectivity values between channels are greater than zero. The greater the values, the stronger the connectivity between channels, and the thicker and more opaque the line. In contrast, black-and-white lines mean the connectivity values are less than zero. Similarly, the smaller the values, the weaker the connectivity between channels, and the thinner and more transparent the lines.

We calculate the connectivity of between-channel to evaluate the connectivity of between-BR or between-L/RH. For *n* BRs(or L/RHs) *z* = {*z*_1_, *z*_2_, ⋯ , *z*_*k*_, ⋯ , *z*_*n*_}, ∃*z*_*a*_∈*z* and ∃*z*_*b*_∈*z* (*a, b*∈[1, *n*], *a*≠*b*). For *N* channels {*x*_1_, ⋯ , *x*_*i*_, ⋯ , *x*_*N*_} and *M* channels {*y*_1_, ⋯ , *y*_*j*_, ⋯ , *y*_*M*_}, ∀*x*_*i*_∈*z*_*a*_ and ∀*y*_*j*_∈*z*_*b*_ (*i*∈[1, *N*], *j*∈[1, *M*]). *C*(*x*_*i*_, *y*_*j*_) represents the connectivity between channel *x*_*i*_ and channel *y*_*j*_ obtained from between-channel connectivity. Therefore, the connectivity *C*(*z*_*a*_, *z*_*b*_) between BR(or L/RH) *z*_*a*_ and BR(or L/RH) *z*_*b*_ can be denoted as:


(7) $$\begin{array}{r} C\left(z_{a},z_{b}\right)=\overset{N}{\underset{i}{\sum }}\overset{M}{\underset{j}{\sum }}C\left(x_{i},y_{j}\right) \end{array}$$

## 4. Experiments

### 4.1. Datasets

We use two different datasets: SEED and Fatigue Driving to verify the performance of EEG-FCV.

#### 4.1.1. SEED Dataset

SEED is a public dataset issued by SJTU for EEG emotion recognition (30, 31). Fifteen Chinese subjects (male:female = 7:8, MEAN: 23.27, STD: 2.37) are recruited to participate in the experiments. Each subject is asked to perform the experiment three times with an interval of approximately 1 week, and watch fifteen Chinese film clips with three emotion stimuli (positive, neutral, and negative emotions) in each experiment. The duration of each film clip is approximately 4 min. There is a total of 15 trials for each experiment. EEG is recorded by EEG cap with 62 channels in line with the international 10–20 system. Then EEG is downsampled to 200 Hz and filtered with a bandpass frequency from 0 to 75 Hz.

#### 4.1.2. Fatigue Driving Dataset

The fatigue driving dataset includes 20 healthy subjects (9 women, 11 men) of EEG data collected in 2 h of driving for mental state prediction. Each subject is required to perform the driving tasks twice on 2 consecutive days. The experiment is conducted following the principles outlined in the Declaration of Helsinki of 1975, as revised in 2008.

EEG is recorded with a sampling frequency of 200 Hz by a 62-channel EEG cap (Brain Products GmbH, Munich, Germany), then EEG data between 1 and 30 Hz is remained by band-pass filtering, and eye blinking artifacts are removed by means of Independent Component Analysis (ICA) (32). Fatigue Driving dataset contains EEG of five mental states namely TAV1 to TAV5 evoked by two stimuli of video and audio, as well as the right and left button press. In addition, EEG of the other three mental states (WUP, PERFO, DROWS) is also recorded without any stimuli but at different driving speeds. Please refer to Zeng et al. (33, 34), and Zhao et al. (35) for more details.

### 4.2. Comprehensive vs. PCC, Coherence and PLV

**Figure 5:**
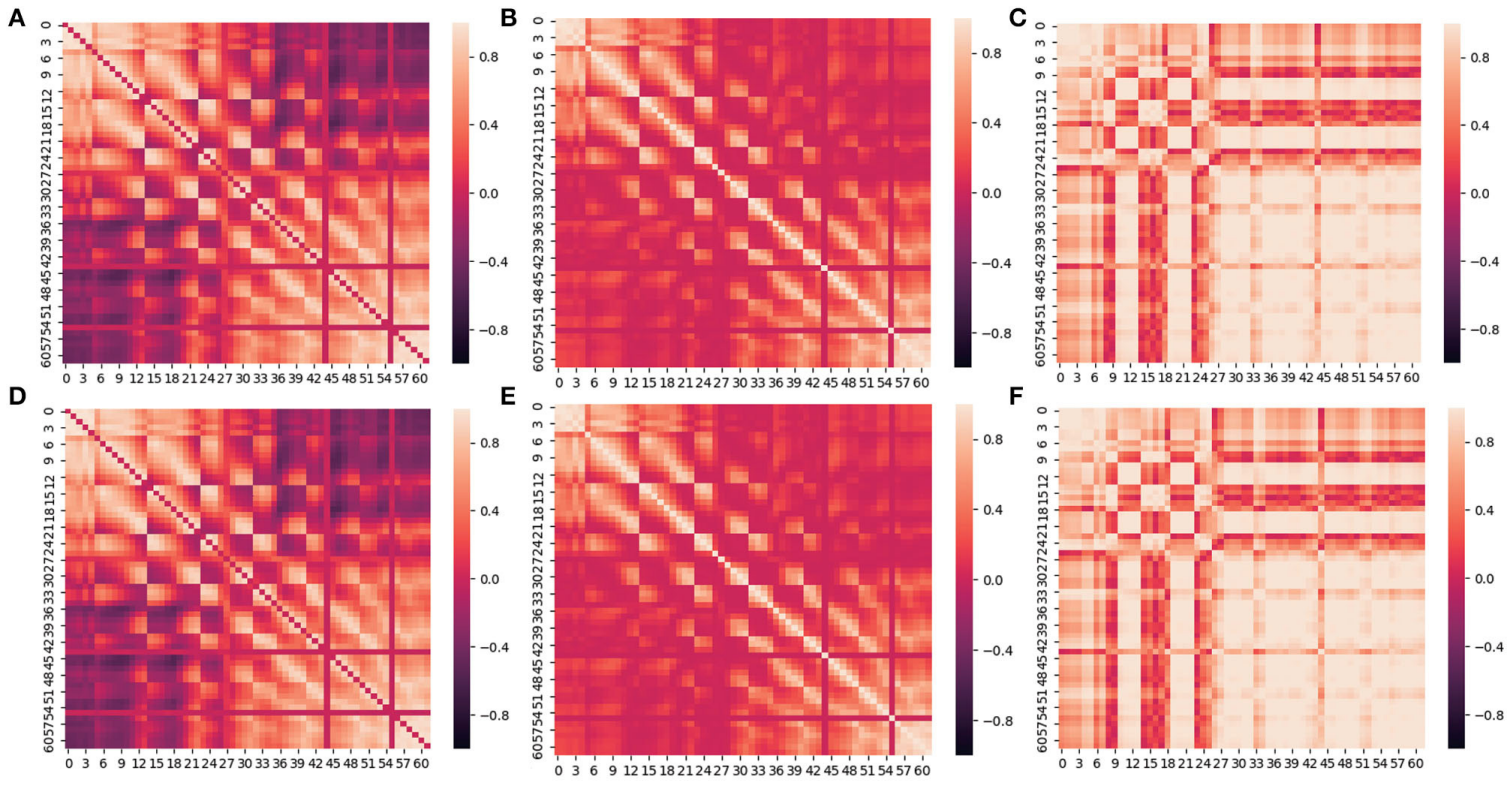
Between-channel FC visualization of PCC and Comprehensive. **(A–C)** is the heat map of PCC, Coherence and PLV. **(D–F)** is the heat map of Comprehensive based on **(A–C)**.

First of all, we compared the results of using different FC metrics. Figure 5 shows the results by means of PCC, Coherence, PLV, and Comprehensive in the EEG alpha band of the SEED dataset.

We can see from Figures 5A–C that the closer the distance between two channels, the larger the value of Coherence, PCC, and PLV, and the stronger the connectivity. Conversely, the farther the distance between two channels, the smaller the value of Coherence, PCC, and PLV, and the weaker the connectivity. The values of PLV between two channels are usually larger than Correlation and PCC. In addition, the connectivity between channels in the frontal lobe(F region), the frontal cortex(FC region), and the frontal temporal lobe(FT region) is commonly stronger than in other regions.

When using Comprehensive to measure and visualize brain FC, as shown in Figures 5D–F, the connectivity between channels with larger Comprehensive is highlighted by assigning a greater positive weight (≥0) to the connection. Correspondingly, the connectivity with smaller Comprehensive between channels is weakened by assigning a smaller negative weight ( ≤ 0) to the connection. Visual clutter of brain FC can be effectively eliminated in this way.

### 4.3. FC Visualization

In this section, we take PCC and corresponding generated Comprehensive based on PCC as examples to show brain FC in 3D in our proposed visualization modules. Similar results can be obtained based on the other two metrics and corresponding Comprehensive metrics.

#### 4.3.1. Between-Channel Visualization

**Figure 6:**
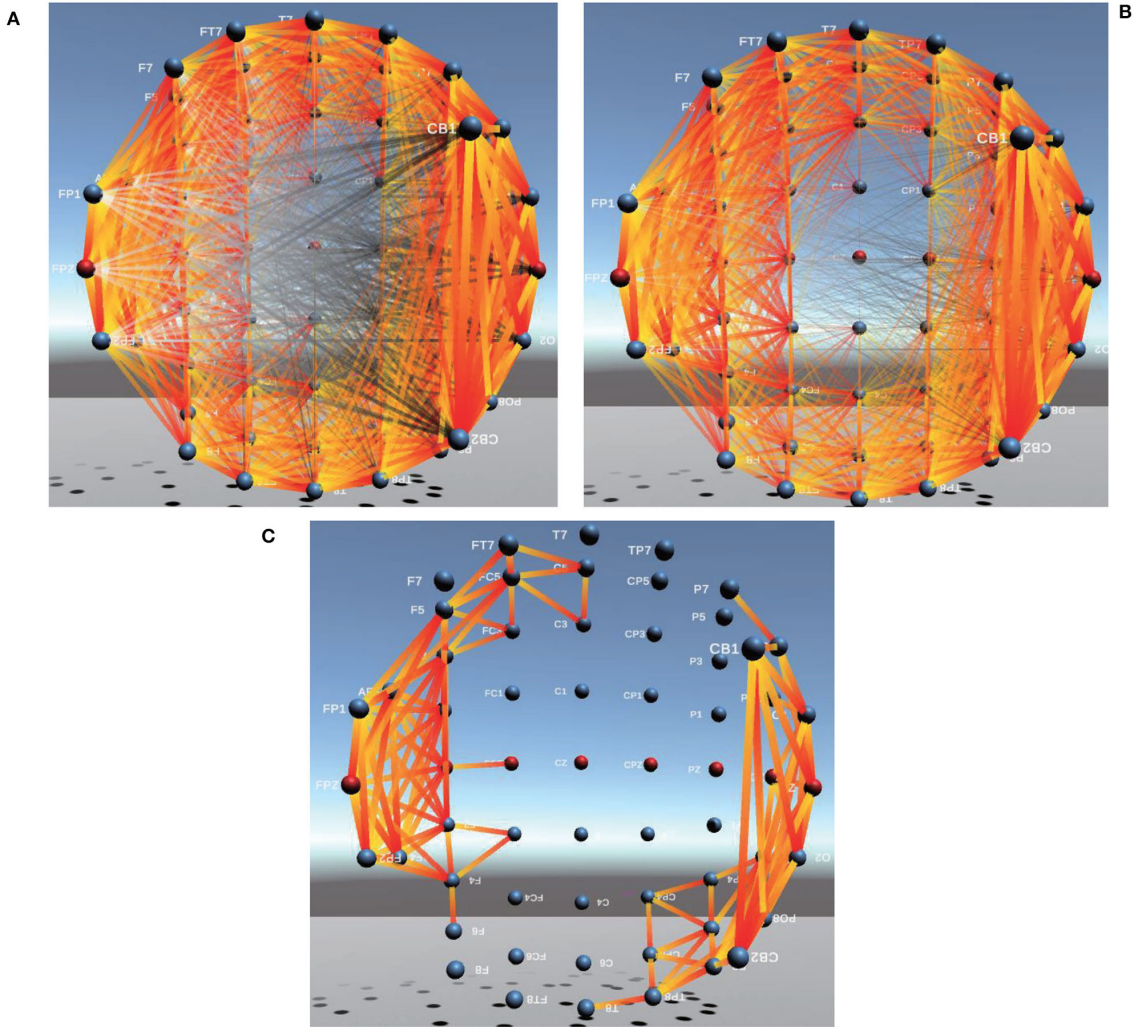
Between-channel FC visualization of PCC and Comprehensive. **(A,B)** are between-channel FC visualization of PCC and Comprehensive. **(C)** is Between-channel FC visualization of 1/8 of Comprehensive.

Brain FC based on PCC in 3D is shown in Figure 6, from which the phenomenon of visual clutter can be easily observed so that it is very difficult to evaluate the connectivity between channels. After adopting Comprehensive, the important connections between brain channels (the corresponding Comprehensive value is larger) become more prominent, and those connections with negative Comprehensive values are faded or even disappeared so that the problem of visual clutter can be effectively alleviated, the visualization of brain FC by Comprehensive is shown in Figure 6B.

In addition, to further eliminate visual clutter, we define four kinds (1/2, 1/4, 1/8, and 1/16) of visualization modes.

To obtain 1/2 of Comprehensive, the mean value of Comprehensive between channels is firstly calculated, then compared with Comprehensive values of each pair of channels. If the mean value of Comprehensive is greater than 0, only those connections whose Comprehensive values are greater than the mean value of Comprehensive are displayed in the visualization of brain FC; If mean value of Comprehensive is less than 0, only those connections whose Comprehensive values are less than the mean value of Comprehensive are displayed in the visualization of brain FC. Similarly, 1/4, 1/8, and 1/16 of Comprehensive can also be obtained. In this way, important connections between channels are highlighted and those minor ones are faded or even ignored so that the problem of visual clutter in visualization can be effectively avoided. The connectivity of brain FC by PCC, Comprehensive, and 1/8 of Comprehensive in 3D is shown in Figures 6A–C, respectively. We can find that those important connections in an analysis of cognitive states are all displayed prominently, and the visualization of brain FC in 3D becomes more clearer than those of PCC in Figure 6A and of Comprehensive in Figure 6B.

In addition, we could also find from Figure 6 that the connectivity strength between two adjacent channels is the largest. The farther the distance between two channels, the lower the connectivity strength. Furthermore, we also find the connectivity strength between channels in the Frontal lobe(F region), Arcuate Fasciculus (AF region), Parieto Occipital (PO region), and Occipital lobe(O region) is usually greater than that in other brain regions.

#### 4.3.2. Visualization Between Brain Regions

Because Comprehensive is commonly used to measure the connectivity between two channels in the same brain region, it is not applicable to measure the connectivity strength between different brain regions and between left and right brain hemispheres. Therefore, we employ PCC to assess the strength of connectivity between different brain regions and between left and right brain hemispheres and then visualize them in 3D.

**Figure 7:**
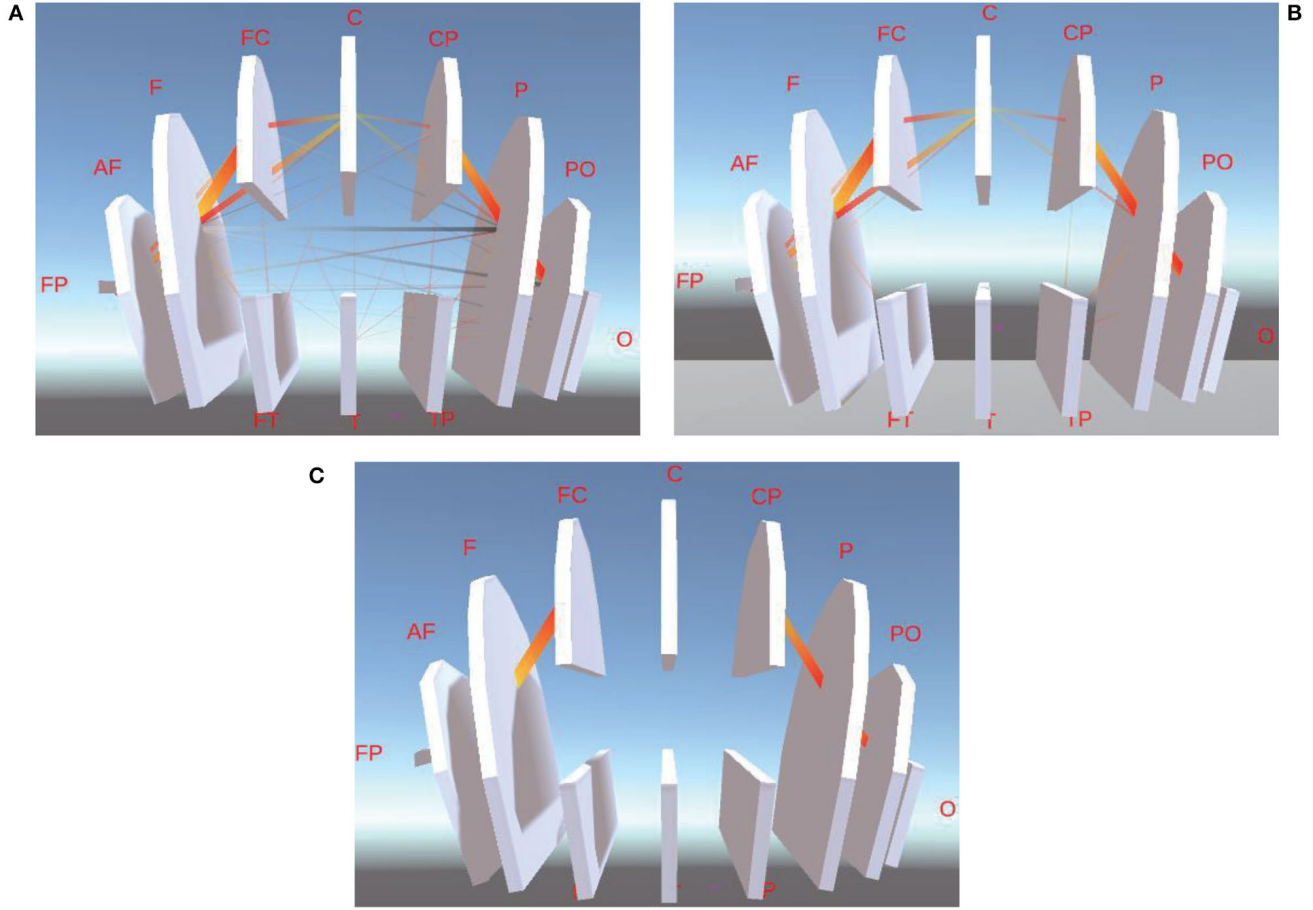
This is between-BR FC visualization of different ranges of PCC. **(A)** is between-BR FC visualization of the whole PCC. **(B,C)** are between-BR FC visualization of 1/2 and 1/8 of PCC.

In addition, we obtain the sum of PCC values of each pair of channels in the same brain region (Figure 7A), called PCC of the brain region, then adopt the same approach in Section 4.3.1 to calculate 1/2, 1/4, 1/8, and 1/16 of PCC of one brain region, for the avoidance of visual clutter in between-BR visualization.

Figures 7B,C are the between-BR connectivity of 1/2 and 1/8 of PCC, respectively. From them, we can learn that the Between-BR connectivity between the Frontal lobe and Frontal Cortex(F-FC), between Central Parietal and Parietal lobe(CP-P), and between the Parietal lobe and Parieto Occipital(P-PO) is much stronger.

#### 4.3.3. Visualization Between Left/Right Hemisphere

**Figure 8:**
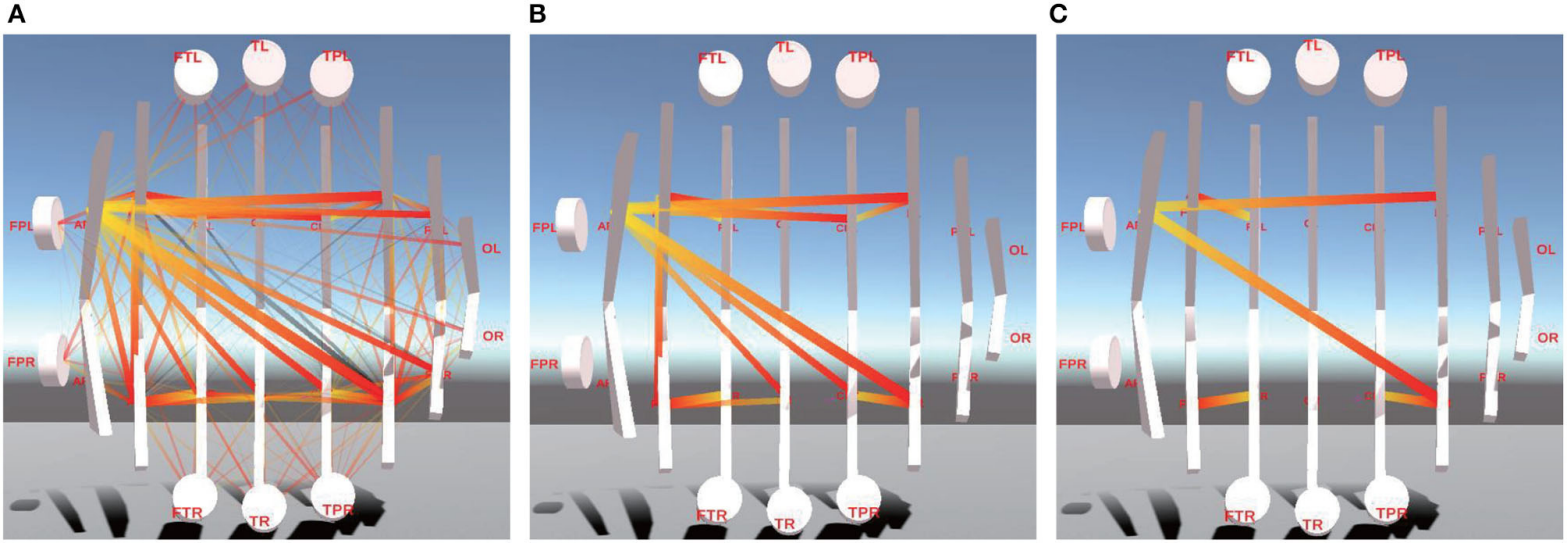
This is between-L/RH FC visualization of different ranges of PCC. **(A)** is between-L/RH FC visualization of the whole PCC. **(B)** is between-L/RH FC visualization of 1/2 of PCC. **(C)** is between-L/RH FC visualization of 1/8 of PCC.

Similar to Section 4.3.1, 1/2, 1/4, 1/8, and 1/16 of PCC between left and right brain hemispheres can also be obtained by summing PCC values of each pair of channels of the left and right brain hemisphere. Figure 8 shows between-L/RH FC connectivity. Figure 8A is the connectivity of PCC of the left and right brain hemispheres. Figures 8B,C show between-L/RH connectivity of 1/2 and 1/8 of PCC.

It is obvious that the connectivity between left Arcuate Fasciculus (AFL region) and Central zone(C region), between Central Parietal (CP region) and Parietal lobe (P region), between left Frontal lobe and right Frontal lobe (FL-FR), and between left Frontal lobe and left Frontal Cortex (FL-FCL) is significantly stronger than connectivity of other L/RHs.

#### 4.3.4. Visualization of Internal Connectivity

Tables 1–3 show the internal-connectivity results of the inter-hemispheres, inter-BR, and inter-L/RH, respectively. Connectivity between channels in the right hemisphere of the brain is slightly stronger than that in the left hemisphere, and the internal connectivity in the frontal lobe (F region) and parietal lobe (P region) is much stronger. In addition, the internal connectivity of the left arcuate fasciculus (AFL region) and right frontal lobe (FR region) is greatly stronger than that of other brain regions.

**Table 1:** Inter-hemisphere FC with PCC.

**Hemisphere**	**PCC**
Left	260.48
Right	278.0063

**Table 2:** Inter-BR FC with PCC.

**BR**	**AF**	**C**	**CP**	**F**	**FC**	**FP**
PCC	0.49	9.45	16.56	46.87	21.71	5.89
**BR**	**FT**	**O**	**P**	**PO**	**T**	**TP**
PCC	–0.46	5.74	31.81	15.48	–0.18	0.55

**Table 3:** Inter-L/RH FC with PCC.

**L/RH**	**PCC**	**L/RH**	**PCC**	**L/RH**	**PCC**
AFL	12.75	FL	9.77	PL	4.88
AFR	0	FPL	0	POL	1.58
CL	3.59	FPR	0	POR	1.79
CPL	4.75	FR	10.07	PR	9.07
CPR	4.53	FTL	0	TL	0
CR	4.16	FTR	0	TPL	0
FCL	4.68	OL	0	TPR	0
FCR	4.95	OR	9.77	TR	0

#### 4.3.5. Visualization of Comparable Connectivity

Comparable Connectivity Visualization is to analyze brain FC and its differences under different cognitive states (i.e., different emotions). Similarly, the comparable FC of 1/2, 1/4, 1/8, and 1/16 of PCC can also be obtained by summing PCC between channels.

**Figure 9:**
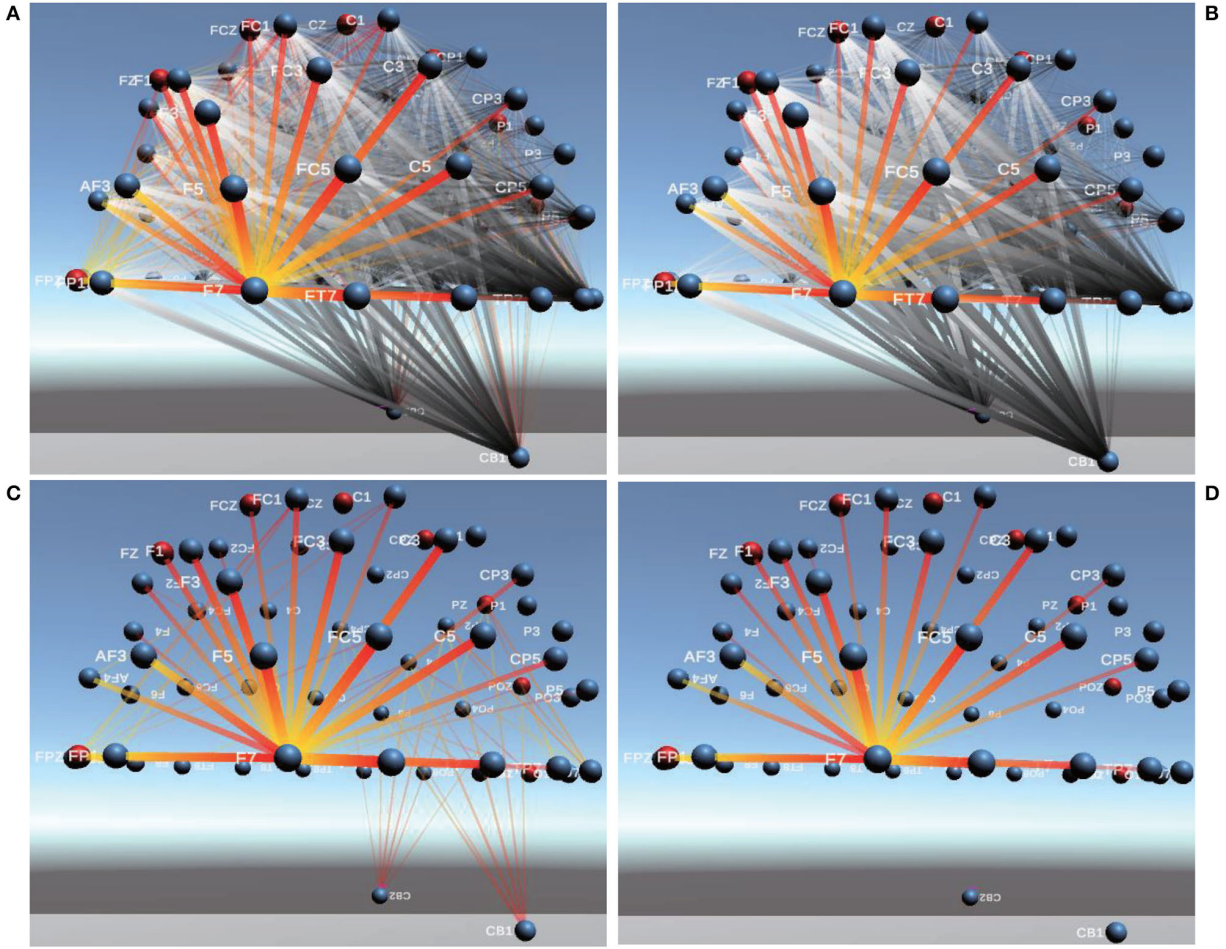
Comparable Connectivity Visualization of PCC and Comprehensive. **(A,B)** are Comparable Connectivity Visualization of PCC and Comprehensive. **(C,D)** are Comparable Connectivity Visualization of 1/2 of PCC and Comprehensive.

For example, we perform the connectivity comparison analysis of two emotional states: neutral and positive by using PCC and Comprehensive, as shown in Figure 9, gold-and-red lines indicate stronger connectivity than the contrast experiment. The greater the difference between the connectivity of the two recognitive states is, the thicker the line indicating connectivity is and the more opaque it is. Among them, Figure 9A shows comparable FC of PCC between channels. It can be clearly seen that the connectivity of neutral emotions is stronger than that of positive emotions near channel F7. In addition, the connectivity between other channels is weaker than that of positive emotions. Figure 9B shows comparable FC of Comprehensive between channels. The connectivity of neutral emotions becomes much more invisible for CB1 and CB2. Figures 9C,D show comparable FC of 1/2 of PCC and Comprehensive, indicating Comprehensive performs better than PCC in avoiding visual clutter.

#### 4.3.6. Visualization of Dynamic Connectivity

**Figure 10:**
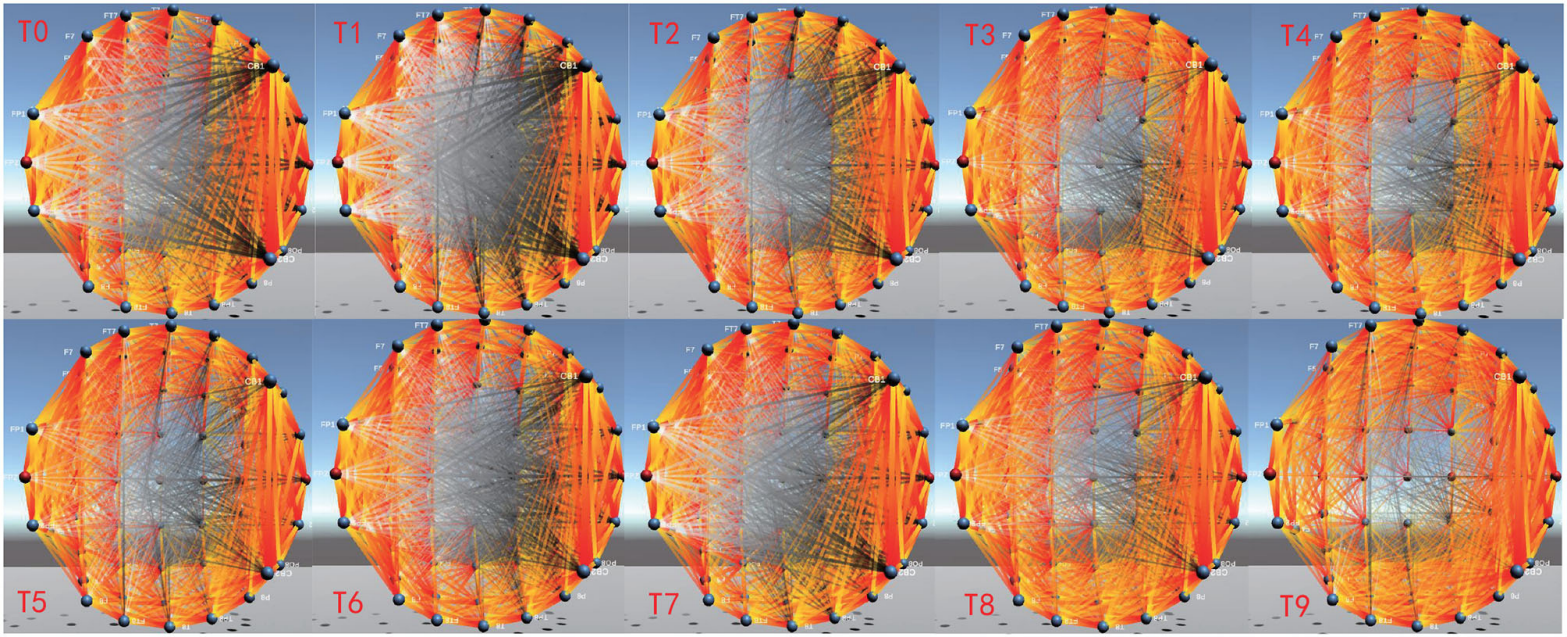
Dynamic visualization changes over time.

EEG-FCV can also display dynamic changes of brain FC over time through Dynamic Connectivity, which provides assistance for the dynamic evolution analysis of brain cognitive function. Figure 10 shows the FC dynamic visualization in the EEG alpha band from time *T*0 to *T*9, which is implemented by constructing a corresponding dynamic functional connectivity matrix based on PCC. Compared with the connectivity between two channels that is farther apart, the connectivity between two adjacent channels has more obvious changes over time.

## 5. Discussion

We design and implement EEG-FCV to analyze the connectivity of 15 participants in the SEED dataset. From the experimental results, we can further confirm the connectivity between adjacent channels is generally stronger than that between non-adjacent (also called distant) channels. Furthermore, we verify the findings of the connectivity in the frontal lobe (F region) and the arcuate fascicle (AF region) are stronger, especially in the left arcuate fascicle (AFL), as Kim found in 2007 (36). In addition, as Dasdemir found in 2017 (37), we also find that interhemispheric connectivity is weaker than intra-hemispheric connectivity.

We compare the connectivity changes of different metrics in different frequency bands. First, we find that when subjects are stimulated with negative emotion, the right hemisphere is more active than the left hemisphere, which is consistent with the results found by Alfano and Cimino (38). Therefore, positive emotions are activated relative to the left hemisphere (as measured by a reduction in the alpha band) and negative emotions are activated relative to the right hemisphere as Ahern pointed out in 1985 (39). It is known that alpha oscillatory activity plays an important role in cognitive processing (40). Our findings confirm the alpha band oscillations because the alpha bands are much more tightly connected than the electrodes in the beta band and delta band.

In the fatigue driving dataset, DROW is the most boring and monotonous task without any stimulation after nearly 2 h of driving, which can be prone to make the subject tired. While in TVA3, as a driving task with medium difficulty, the subject drives his car accompanied by video and audio stimuli, so that he is in an alert state. Therefore, we compare the connectivity differences in different frequency bands by different functional connectivity metrics between TVA3 and DROW states, respectively. We find, as we predict, that the connectivity of subjects is generally stronger during TVA3 than in the state of fatigue. Similar to the findings of Kakkos et al. (41), we also find that brain regions show much tight connection and more active interaction as workloads increase. In addition, the connectivity between the frontal lobe (F region) and the parietal lobe (P region) is generally stronger than that in other brain regions, which is also mentioned by Corbetta and Shulman (42). And the connectivity between the frontal lobe (F region) and the left arcuate fascicle (AFL region) is significantly stronger than that in other brain regions. This proves once again that the arcuate fascicle (AF region) is thought to provide connectivity for spatial attention, which is consistent with the findings of other researchers (43–46). At the same time, we find that there is a certain increase in the central regional (channel CZ) connectivity during the state of fatigue as charbonnier found in 2016 (47).

Compared with some current tools, such as EEGLab and EEGNet, EEG-FCV has realized the EEG-based analysis of brain FC and implemented the dynamic visualization in 3D with the avoidance of the visual clutter. However, EEG-FCV does not contain the subsequent statistical validation phase, which will be improved in the follow-up study to make it have better versatility in brain connectivity analysis. All full names and abbreviations are described in Table 4.

**Table 4:** Full name and abbreviation.

**Full name**	**Abbreviation**	**Full name**	**Abbreviation**
functional connectivity	FC	between-brain-region	between-BR
2 dimensions	2D	between-left-right hemisphere	between-L/RH
3 dimensions	3D	Independent component analysis	ICA
Pearson correlation coefficient	PCC	frontal cortex	FC
Phase locking value	PLV	frontal temporal	FT
functional magnetic resonance imaging	fMRI	arcuate fasciculus	AF
diffusion tensor imaging	DTI	parieto occipital	PO
Supplementary motor area	SMA	central parietal	CP
Primary motor area	MI	left arcuate fasciculus	AFL
maximal clique-based	MCB	left frontal	FL
watershed-based	WB	left frontal cortex	FCL
Wavelet packet decompotion	WPD	right frontal	FR

## 6. Conclusion

In this study, we design an EEG-based cognitive function visualization system, EEG-FCV, and validate it on the SEED emotion dataset and fatigue driving dataset. The results show that EEG-FCV can effectively display the visualization effect of functional connectivity of EEG data in different frequency bands using PCC, correlation, and PLV. In order to solve the problem of visual clutter, we also propose a novel metric of Comprehensive, which can effectively display the difference in connectivity results and visualize brain FC much more clearly. We believe EEG-FCV can be useful in promoting EEG-based brain FC analysis and visualization.

## Data Availability Statement

Publicly available datasets were analyzed in this study. This data can be found here: https://bcmi.sjtu.edu.cn/home/seed/downloads.html; https://www.mdpi.com/1424-8220/21/7/2369/.

## Author Contributions

HZ: writing-review, editing, supervision, and funding acquisition. YJ: conceptualization, methodology, software, and writing-original draft. QW: writing-original draft. DP and FX: validation. YZ: project administration. HH: supervision. WK: supervision and funding acquisition. All authors contributed to the article and approved the submitted version.

## Funding

This study is partly supported by the National Key R&D Program of China with Grant No. 2017YFE0116800 and partly supported by NSFC with Grant No. 62076083. The authors also thank the National International Joint Research Center for Brain-Machine Collaborative Intelligence with Grant No. 2017B01020, Key Laboratory of Brain Machine Collaborative Intelligence of Zhejiang Province with Grant No. 2020E10010.

## Conflict of Interest

The authors declare that the research was conducted in the absence of any commercial or financial relationships that could be construed as a potential conflict of interest. The handling editor declared a past collaboration with one of the authors HZ.

## Publisher's Note

All claims expressed in this article are solely those of the authors and do not necessarily represent those of their affiliated organizations, or those of the publisher, the editors and the reviewers. Any product that may be evaluated in this article, or claim that may be made by its manufacturer, is not guaranteed or endorsed by the publisher.
